# Clinical treatment of postoperative infection following sinus augmentation

**DOI:** 10.5051/jpis.2010.40.3.144

**Published:** 2010-06-25

**Authors:** Seung-Bum Hong, Jae-Suk Kim, Seung-Il Shin, Ji-Young Han, Yeek Herr, Jong-Hyuk Chung

**Affiliations:** 1 Gangnam Hyundai Dental Clinic, Seoul, Korea.; 2 Luden Dental Clinic, Seoul, Korea.; 3 Department of Periodontology, Kyung Hee University School of Dentistry, Seoul, Korea.; 4 Department of Dentistry/Periodontology, Hanyang University School of Medicine, Seoul, Korea.; 5 Department of Periodontology, Institute of Oral Biology, Kyung Hee University School of Dentistry, Seoul, Korea.

**Keywords:** Dental implants, Maxillary sinus, Surgical wound infection

## Abstract

**Purpose:**

The aim of this case report is to present the successful clinical treatment of two cases of postoperative infection following maxillary sinus augmentation.

**Methods:**

In the two cases of postoperative infection, immediate total removal of the grafted material from the sinus was conducted to stop the spread of the infection, after which a high dose of antibiotics was administrated. Re-augmentation procedures were then conducted after the infection subsided.

**Results:**

No further complications occurred after sinus re-augmentation. The dental implants placed in the re-augmented sinus were clinically osseointegrated, and the implant-supported restorations in the two cases of postoperative infection have been functioning very well for over 2 years.

**Conclusions:**

In the case of infection of the grafted sinuses, it is necessary to completely remove the graft materials and then administer a high dose of antibiotics to treat the acute infection, after which sinus re-augmentation is suggested.

## INTRODUCTION

Osseointegrated dental implant has become a predictable and effective clinical procedure [1]. Although many authors have reported high success rates for dental implants [2,3], problems often occur during reconstruction of the posterior maxilla using implant-supported prostheses. For example, alveolar bone loss following tooth extraction and increased pneumatization of the maxillary sinus reduce the quantity of residual bone necessary to place the dental implants. Boyne and James [4] first reported grafting of the maxillary sinus floor with autogenous marrow and bone. They described grafting the sinus by conducting an antrostomy on the lateral antral wall that was, approximately 1 cm in diameter, after which the antral membrane was elevated using a modified curet-like instrument. The space under the elevated antral membrane was then filled with an autogenous graft. Many authors have modified the original surgical procedures and used various graft materials [5-7]. Indeed, improvement of the implant surface has led to increased survival and success rates during procedures conducted to augment the maxillary sinus [8]. Although high success rates have been reported for implants placed in the augmented sinus, clinicians have experienced various complications, including perforation of the sinus membrane, maxillary cyst, excessive bleeding, infection of the grafted sinuses, and failure of the bone formation during and after sinus augmentation [9-17]. Among these complications, infection of the grafted material may spread to the orbita or even to the brain [14]. Accordingly, immediate treatment should be conducted to stop the infection from spreading. However, because infection of the grafted sinus is less common, there are few clinical reports describing this condition. In this clinical case report, the causes and managements of postoperative infection after sinus augmentation are presented. The management techniques include surgical removal of the infected graft materials, administration of a high dose of antibiotics to treat the infection and sinus re-augmentation after the infected sinus has healed.

## CASES DESCRIPTION

### Case 1

**Figure 1:**
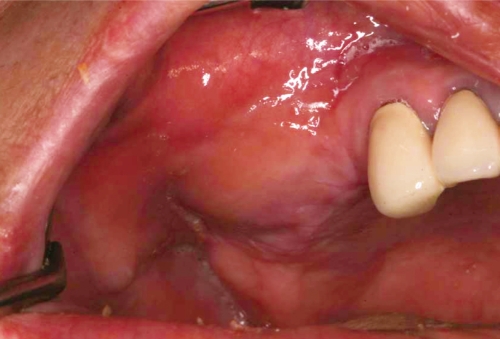
Postoperative infection following sinus augmentation.

**Figure 2:**
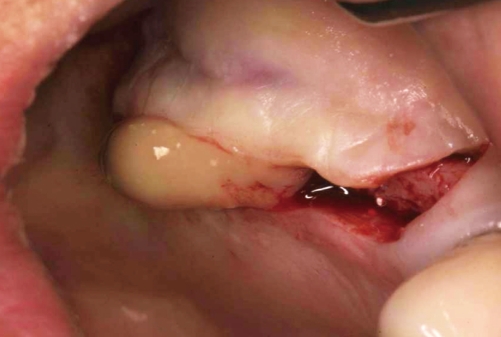
The discharge of pus and graft materials after the incision.

**Figure 3:**
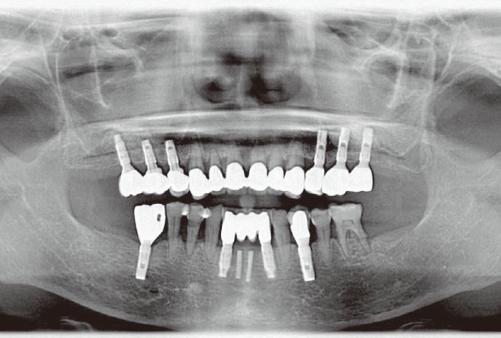
Postoperative panoramic radiograph following sinus re-augmentation along with the placement of dental implants.

A 60-year-old female visited the clinic for reconstruction of both sides of the edentulous posterior maxilla. Radiographic examination revealed large pneumatization of both maxillary sinuses. The residual bone height was between 2 mm and 5 mm. Augmentation of both maxillary sinuses was scheduled to be conducted first, followed by placement of the dental implants 9 months later. However, the sinus membrane of the left side was very difficult to elevate due to the presence of what was suspected to be pus in the inner portion of the left sinus membrane. After confirming the presence of pus by aspiration, complete removal of the pus was conducted by intentional perforation of the sinus membrane. The sinus augmentation was then performed after repairing the intentionally perforated portion with absorbable collagen wound dressing (CollaTape®, Zimmer Dental Inc., Carlsbad, USA). However, the right sinus was clinically healthy. Both sinuses were then grafted with deproteinized bovine bone (Bio-Oss®, Geistlich AG, Wolhusen, Switzerland). The possibility of postoperative infection of the left sinus was explained to the patient after the augmentation procedure. However, the patient visited the clinic 3 weeks later due to swelling of the right posterior maxillary area (Figs. 1 and 2). Immediate removal of the graft materials from the sinus was conducted and saline irrigation was performed several times. Antibiotics (Augmentin 375 mg, Ilsung Pharmaceuticals, Seoul, Korea) were prescribed three times a day for 7 days and 0.12% chlorhexidine solution (Hexamedine, Bukwang Pharmaceutical, Seoul, Korea) was also prescribed twice a day for the first 2 weeks. After removal of the graft materials and prescription of the antibiotics, the infected sinus was successfully treated. The right maxillary sinus was then re-augmented 9 months after postoperative infection due to the patient's schedule. At that time, the inner portion of the buccal flap had fused with the sinus membrane. The membrane had also clinically hardened and thickened, with some portions containing residual graft particles. The removal of the graft was impossible because the membrane and the residual graft had fused together. Therefore, the sinus membrane was separated from the inner portion of the flap by sharp dissection and the stiffened membrane was then carefully elevated. Due to the thickened and hardened condition of the sinus membrane, membrane perforation seldom occurred during re-augmentation. Dental implants were then placed 7 months after sinus re-augmentation. No further complications were observed following re-operation and final restorations were made 10 months later. The implants placed in the re-augmented sinus were clinically healthy and the implant-supported restorations have been functioning successfully for 26 months (Fig. 3).

### Case 2

**Figure 4:**
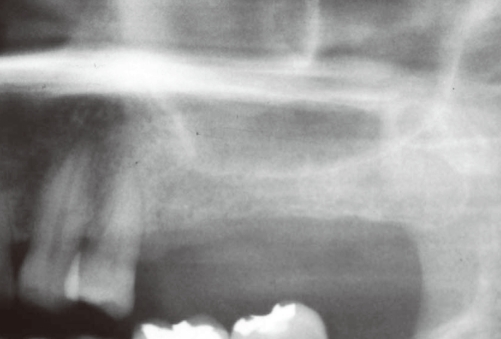
Pneumatization on the left maxillary sinus was observed in the preoperative panoramic radiograph.

**Figure 5:**
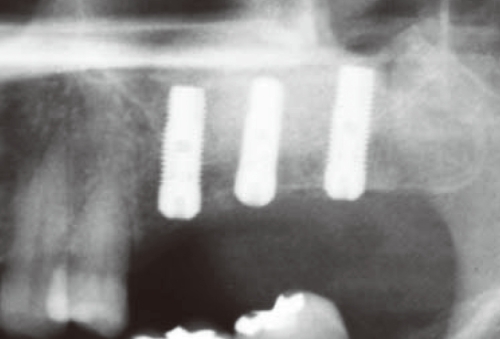
Implant placed on positions #25-27 simultaneously with the sinus augmentation.

**Figure 6:**
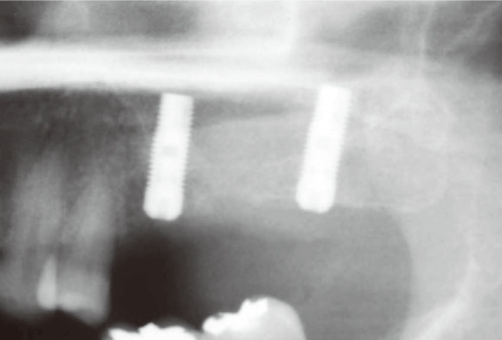
Infected graft material was surgically removed and #26i was removed to ease removal of the infected graft.

**Figure 7:**
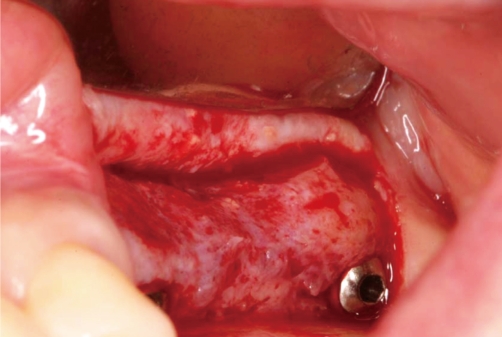
The inner portion of the buccal flap and the sinus membrane were fused. The membrane and the residual graft material were inseparably fused together.

**Figure 8:**
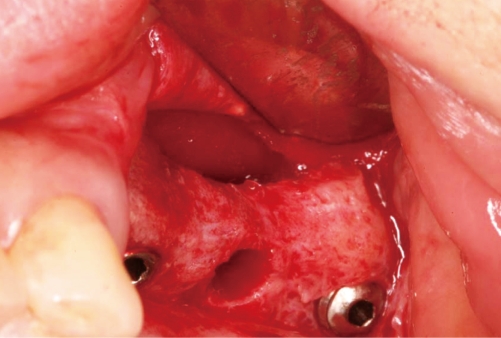
Elevation of the sinus membrane and ostectomy were performed.

**Figure 9:**
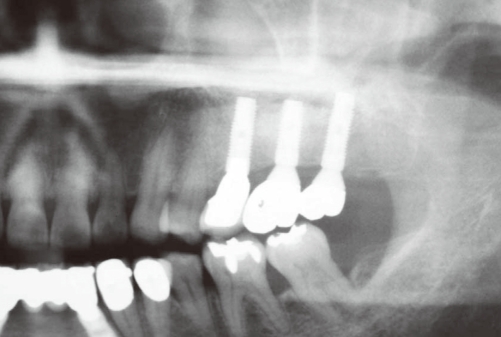
Panoramic radiograph of the dental implants placed in the re-grafted sinus after successfully functioning for 33 months.

A 49-year-old female visited the clinic for reconstruction of the left edentulous posterior maxilla. Radiographic examination revealed that the available bone height was approximately 4-5 mm (Fig. 4). The dental implants were placed simultaneously with sinus augmentation because there was sufficient residual bone for the primary stability of the dental implants. The maxillary sinus was then grafted with deproteinized bovine bone and three Astra Tech implants (Tioblast®, Astra Tech Dental Implant System, Mölndal, Sweden) 4 mm in diameter and 13 mm in length were placed at the site of #25i-#27i (Fig. 5). Eight days after the procedure, the patient complained of swelling and pain at the surgical area. Evaluation revealed that the left buccal vestibule was swollen due to pus formation. Under local anesthesia, an incision was made at the surgical area to drain the pus and infected grafted materials from the maxillary sinus, after which antibiotics (Augmentin 375 mg, Ilsung Pharmaceuticals, Seoul, Korea) were prescribed three times a day for 7 days and 0.12% chlorhexidine solution (Hexamedine, Bukwang Pharmaceutical, Seoul, Korea) was also prescribed twice a day for the first 2 weeks. The infected sinus was successfully treated after the pus was drained and the graft material was completely removed from the sinus. Although the implant at the #26i site was explanted to facilitate the removal of the graft material, the implants at #25i and #27i were left in place (Fig. 6). Sinus re-augmentation was then conducted 3 months later, at which time the implant was replaced simultaneously at position #26i (Figs. 7 and 8). When compared to case 1, the sinus membrane was less stiffened and hardened. This difference may have been due to the difference in waiting period before the sinus re-augmentation. Consequently, membrane elevation in this case was easier to perform than in case 1. After exposing portions of the dental implants at the site of #25i and #27i, the site was rinsed with tetracycline-HCl solution (50 mg/mL) several times to sterilize the area, after which deproteinized bovine bone mixed with tetracycline (50 mg/mL) was grafted simultaneously with the placement of the implant at #26i. No further complications occurred following sinus re-augmentation, and the implants were loaded 8 months later. The dental implants that were placed in the re-grafted sinus have been functioning very well for 33 months (Fig. 9).

## DISCUSSION

This case report demonstrates that surgical procedures that involve total removal of the grafted material, prescription of antibiotics, and sinus re-augmentation can be used to successfully treat postoperative sinus infection. Although sinus augmentation is very predictable [8] and complications caused by sinus graft are very rare, clinicians have experienced various types of complications, such as perforation of the sinus membrane, excessive bleeding, infection of the grafted sinuses, and failure of the formation of bone during and after sinus augmentation procedures [9-17]. Among these complications, infection of the grafted sinuses is less common, but if it occurs, the infection can spread quickly to the adjacent areas resulting in brain abscess, infraorbital abscess, and orbital cellulites [14]. For the above reasons, infected sinuses should be treated immediately. According to Anavi et al. [18], postoperative complications often occur due to a poor preoperative clinical status. Misch [19] reported that factors contributing to the development of the sinus infection included perforation of the sinus membrane, inoculation of the graft with saliva, dehiscence of the incision line, and lack of aseptic conditions.

Barone et al. [15] described seven maxillary sinus augmentation procedures performed in 7 patients who exhibited suppuration 3 to 5 weeks after surgical treatment. Five of those seven patients were smokers. The sinus infections in those patients were treated by draining the area through the bony window and subsequent administration of systemic antibiotics. Five patients received additional sinus augmentation 4 to 6 months after the sinus suppuration. Schwartz-Arad et al. [16] investigated 81 cases involving 70 patients and found only 1 report of infection. In the infected case, curettage and H_2_O_2_ irrigation were used to treat the infected area. Membrane perforations appear to be associated with postoperative complications following sinus augmentation.

Zijderveld et al. [17] reported infection after sinus augmentation along with a purulent discharge in 2 patients with local wound dehiscence. These patients were treated with antibiotics and local debridement. Lindhe et al. [20] found that surgical removal of all of the graft material from the sinus cavity and subsequent administration of high doses of antibiotics was essential to the successful treatment of infection. Based on a review of the studies presented above, there are two possible methods of treating such infections. These include: 1) drainage and the administration of systemic antibiotics; 2) total removal of the infected graft and the administration of systemic antibiotics. In this case report, complete removal of the infected graft material was conducted to remove the residual source of infection and treat the infected sinus immediately.

Bravetti et al. [21] reported that the membrane remained healthy after sinus augmentation procedures conducted using graft materials. Similarly, Sul et al. [22] reported that surgical procedures had little effect on the histologic characteristics of the sinus membrane. However, to the best of the author's knowledge, there have been few reports conducted to evaluate clinical and histological changes in the infected sinus membrane following sinus augmentation. In the cases described here, the sinus membrane was clinically different during re-augmentation when compared to the membrane that was subjected to the first sinus augmentation procedure. This was because the sinus membrane that had gone through sinus infection had fused with the inner portion of the buccal mucoperiosteal flap. These findings indicate that, when sinus re-augmentation was applied, reflection of the mucoperiosteal flap should be performed very carefully. If needed, sharp dissection of the fused portion should be conducted for the reflection of the buccal flap without tearing the sinus membrane. Additionally, in the case described here, the membrane elevation procedure was difficult because the sinus membrane had thickened and hardened and some portions contained residual graft particles. It was impossible to remove the residual particles because they had fused with the healed membrane. However, it is believed that these particles played no role in the infection because no further complications were reported following sinus re-augmentation. The differences in the membrane following augmentation are believed to play a role in the negative effects on the mucociliary function of the respiratory epithelium. However, in this case report, the patients did not show any postoperative complications, and the implant-supported restorations have functioned properly for more than 2 years.

Removal of the dental implant in cases of infection should be seriously considered when the infection occurs in the case of simultaneous augmentation and implantation. In case 2, the implant placed at position #26i was explanted to facilitate removal of the infected graft material, but the implants at #25i and #27i were retained. When the infection subsided, the exposed implant surfaces at #25i and #27i were rinsed with tetracycline-HCl solution to sterilize the area. Deproteinized bovine bone mixed with tetracycline was then grafted and the implant was simultaneously placed on #26i. The dental implants at positions #25i and #27i that were exposed to the postoperative infection have been functioning successfully for 33 months without any complications. Therefore, the removal of the dental implants has to be performed selectively, for example, to ease of the removal of an infected graft.

Although postoperative infections related to sinus augmentation are uncommon, they can be very problematic to both patients and clinicians when they do occur. To minimize the occurrence of postoperative infection, the possible causes should be removed prior to sinus augmentation. However, uncontrollable postoperative infection can occur for unknown reasons. The treatments described in this case report consisted of surgical removal of the infected graft material from the sinus and the administration of high doses of antibiotics. These clinical approaches can be used to successfully treat an infected sinus following sinus augmentation.

## References

[1] Albrektsson T (1988). A multicenter report on osseointegrated oral implants. J Prosthet Dent.

[2] Engquist B, Bergendal T, Kallus T, Linden U (1988). A retrospective multicenter evaluation of osseointegrated implants supporting overdentures. Int J Oral Maxillofac Implants.

[3] Jemt T, Lekholm U, Adell R (1989). Osseointegrated implants in the treatment of partially edentulous patients: a preliminary study on 876 consecutively placed fixtures. Int J Oral Maxillofac Implants.

[4] Boyne PJ, James RA (1980). Grafting of the maxillary sinus floor with autogenous marrow and bone. J Oral Surg.

[5] Misch CE (1987). Maxillary sinus augmentation for endosteal implants: organized alternative treatment plans. Int J Oral Implantol.

[6] Summers RB (1994). The osteotome technique: Part 3--Less invasive methods of elevating the sinus floor. Compendium.

[7] Fugazzotto PA, De PS (2002). Sinus floor augmentation at the time of maxillary molar extraction: success and failure rates of 137 implants in function for up to 3 years. J Periodontol.

[8] Wallace SS, Froum SJ (2003). Effect of maxillary sinus augmentation on the survival of endosseous dental implants. A systematic review. Ann Periodontol.

[9] Mardinger O, Nissan J, Chaushu G (2007). Sinus floor augmentation with simultaneous implant placement in the severely atrophic maxilla: technical problems and complications. J Periodontol.

[10] Hernandez-Alfaro F, Torradeflot MM, Marti C (2008). Prevalence and management of Schneiderian membrane perforations during sinus-lift procedures. Clin Oral Implants Res.

[11] Pikos MA (2008). Maxillary sinus membrane repair: update on technique for large and complete perforations. Implant Dent.

[12] Ardekian L, Oved-Peleg E, Mactei EE, Peled M (2006). The clinical significance of sinus membrane perforation during augmentation of the maxillary sinus. J Oral Maxillofac Surg.

[13] Lockhart R, Ceccaldi J, Bertrand JC (2000). Postoperative maxillary cyst following sinus bone graft: report of a case. Int J Oral Maxillofac Implants.

[14] Misch CE (2008). Contemporary implant dentistry.

[15] Barone A, Santini S, Sbordone L, Crespi R, Covani U (2006). A clinical study of the outcomes and complications associated with maxillary sinus augmentation. Int J Oral Maxillofac Implants.

[16] Schwartz-Arad D, Herzberg R, Dolev E (2004). The prevalence of surgical complications of the sinus graft procedure and their impact on implant survival. J Periodontol.

[17] Zijderveld SA, van den Bergh JP, Schulten EA, ten Bruggenkate CM (2008). Anatomical and surgical findings and complications in 100 consecutive maxillary sinus floor elevation procedures. J Oral Maxillofac Surg.

[18] Anavi Y, Allon DM, Avishai G, Calderon S (2008). Complications of maxillary sinus augmentations in a selective series of patients. Oral Surg Oral Med Oral Pathol Oral Radiol Endod.

[19] Misch CM (1992). The pharmacologic management of maxillary sinus elevation surgery. J Oral Implantol.

[20] Lindhe J, Lang NP, Karring T (2008). Clinical periodontology and implant dentistry.

[21] Bravetti P, Membre H, Marchal L, Jankowski R (1998). Histologic changes in the sinus membrane after maxillary sinus augmentation in goats. J Oral Maxillofac Surg.

[22] Sul SH, Choi BH, Li J, Jeong SM, Xuan F (2008). Histologic changes in the maxillary sinus membrane after sinus membrane elevation and the simultaneous insertion of dental implants without the use of grafting materials. Oral Surg Oral Med Oral Pathol Oral Radiol Endod.

